# High-grade serous cancer of left fallopian tube with right inguinal lymph node enlargement: a case report

**DOI:** 10.3389/fonc.2025.1486688

**Published:** 2025-02-06

**Authors:** Aizhi Zhou, Weiyong Gu, Yumei Yang, Xin Chen, Wenfeng Ye, Mei Wang

**Affiliations:** ^1^ Department of Obstetrics and Gynecology, Shanghai Pudong New Area People’s Hospital, Shanghai, China; ^2^ Department of Pathology, Hospital of Obstetrics and Gynecology, Fudan University, Shanghai, China

**Keywords:** HGSOC, ovarian cancer, lymphadenopathy, metastasis, inguinal lymph node

## Abstract

A 47-year-old woman with a two-month history of right inguinal lymphadenopathy visited Shanghai Pudong New Area People’s Hospital for a biopsy. Histopathological and immunohistochemical analyses revealed a metastasis of high-grade serous carcinoma, likely of gynecological origin. A PET-CT scan identified a tumor in the left adnexa, with no other organ involvement. The patient underwent primary cytoreduction, including laparoscopy, hysterosalpingo-oophorectomy, omentectomy, and resection of the right deep inguinal lymph nodes at the Hospital of Obstetrics and Gynecology. No residual disease was found post-surgery. Pathological examination revealed high-grade serous cancer in the fimbria of the left fallopian tube and left ovary, while the right deep inguinal lymph nodes were negative. The patient received standard chemotherapy (Carboplatin and Taxol) and showed no new lesions after three cycles, as confirmed by imaging.

## Introduction

Ovarian cancer remains the most lethal reproductive system cancer, with an estimated 55,000 new cases and 38,000 deaths in China in 2020 (1). According to the Chinese Cause of Death Surveillance, the crude mortality rate of ovarian cancer increased from 1.42 per 100,000 to 2.87 per 100,000 from 2006 to 2020 (2). Common symptoms in ovarian cancer patients include abdominal discomfort or distension, abdominal or pelvic pain, constipation or early satiety, weight loss, and asthenia. Inguinal lymphadenopathy is a rare initial presentation of ovarian cancer (3). Due to the usual lack of symptoms associated with early-stage disease, most cases are diagnosed only after the cancer has progressed. Epithelial ovarian cancer may metastasize intraperitoneally, lymphatically, or hematogenously (4, 5). Lymphatic dissemination from the ovary typically involves the para-aortic and pelvic areas (6), with an estimated incidence of such lesions ranging between 14% and 70% (7). Metastases to pelvic lymph nodes are less frequent than those in the para-aortic region (8–10).

The exact mechanism by which tumor cells spread to the inguinal lymph nodes remains unclear. One possibility is that tumor cells may follow the round ligament of the uterus (11) or the external iliac artery and vein. However, it has been argued that spread to the inguinal lymph nodes should only occur after the blockage of pelvic and para-aortic lymph node stations (12). Considering the peculiarity of this route of spread, the aim of this report is to describe a rare case of high-grade serous cancer of the left fallopian tube with right inguinal lymph node enlargement, with the right deep inguinal lymph nodes negative.

## Case presentation

The patient presented with an enlarged right inguinal lymph node for at least 2 months, which was evaluated at another medical institution. She reported no other symptoms apart from occasional dull pain in the lower abdomen over the past two months. Her medical history included chronic nephritis in 2014 and the resection of a benign cervical vertebral tumor in 2021. Her family history was negative for malignancy, and she had no known occupational or environmental exposure to hormones, particularly estrogens. Clinical examination confirmed the enlarged right inguinal lymph node, which was further evaluated by abdominal ultrasound. The result revealed a heterogeneous solid mass with intrinsic vascularization, measuring 28 × 19 mm. It also showed several smaller reactive lymphadenopathies locoregionally but was otherwise normal. Laboratory analysis was normal except for an elevated CA 125 level of 46 kU/L.

**Figure 1:**
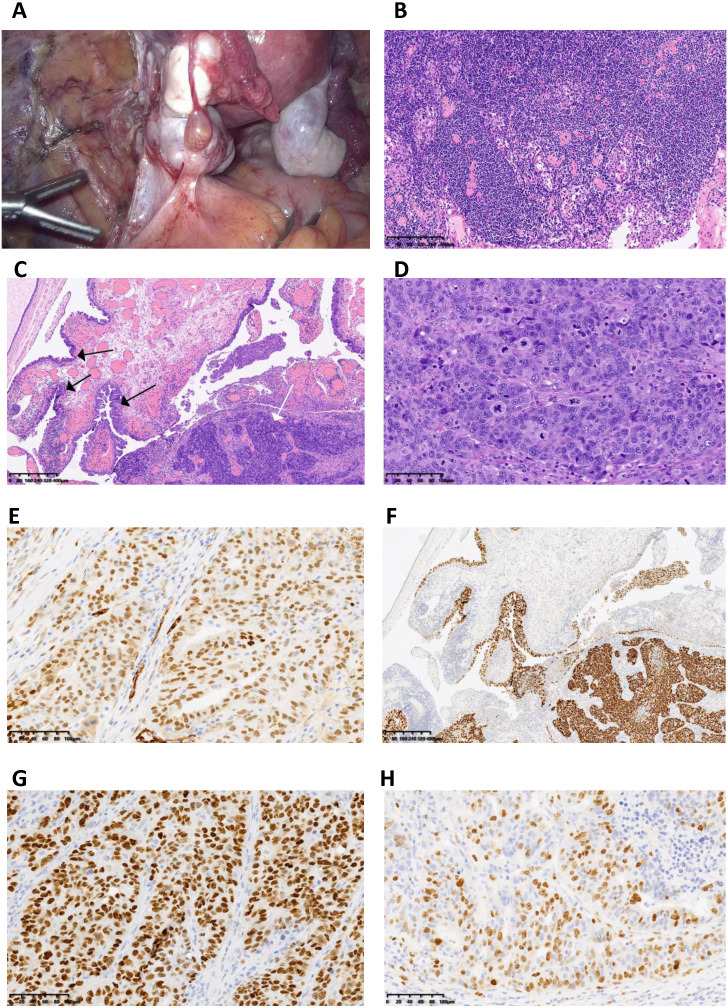
Information of the cancer. **(A)** A solid mass on the surface of the left ovary (size 30×30×25mm). **(B)** No cancer metastasis observed in the right deep inguinal lymph nodes. **(C)** Serous intraepithelial carcinoma (STIC) (indicated by the black arrow) and invasive carcinoma (indicated by the white arrow) observed in the left fallopian tube fimbria. **(D)** High-grade serous carcinoma of the left ovary with mitotic figures observed. **(E)** Immunohistochemical staining shows positive expression of WT1 in the high-grade serous carcinoma of the left ovary. **(F)** Immunohistochemical staining shows diffuse strong positive mutant-type expression of p53 in the lesion of the left fallopian tube fimbria. **(G)** Immunohistochemical staining shows diffuse strong positive mutant-type expression of p53 in the high-grade serous carcinoma of the left ovary. **(H)** Immunohistochemical staining shows a Ki67 proliferation index of 60% in the high-grade serous carcinoma of the left ovary.

A lymph node resection at the first institution revealed metastasis of high-grade serous cancer, likely from the reproductive system. A PET-CT was requested to check for other abnormalities. It confirmed a likely malignant tumor in the left adnexa, showed postoperative fluid accumulation in the right inguinal region, and revealed no other suspicious lesions or ascites. Laparoscopy showed a solid mass on the surface of the left ovary, measuring 30 × 30 × 25 mm (**Figure 1A**). The right ovary and bilateral fallopian tubes appeared normal, and no other macroscopic lesions were found in the abdominal and pelvic cavities (**Figure 1B**). There were no enlarged lymph nodes in the para-aortic or pelvic areas, and no ascites. The pathological examination revealed high-grade serous cancer in the fimbria of the left fallopian tube, which involved the left ovary, but the right deep inguinal lymph nodes were negative (**Figure 1C–H**). Germline and somatic BRCA1/2 testing showed no BRCA1 or BRCA2 mutations. The homologous recombination deficiency (HRD) status was positive, with a score of 53.44 (threshold value: 30).

According to the NCCN guidelines updated in 2024, the patient underwent six cycles of chemotherapy with Carboplatin and Taxol, administered every three weeks for the first four cycles. Bevacizumab was added to the last two cycles as her chronic nephritis was stable and her urine protein turned negative. She was subsequently placed on maintenance therapy with bevacizumab. After four cycles of chemotherapy, imaging showed no lesions. The initially elevated CA 125 level (46 kU/L) declined to 11 kU/L and remained stable.

## Discussion

In this report, we described a rare case of high-grade serous cancer of the left fallopian tube with right inguinal lymph node enlargement, with the right deep inguinal lymph nodes negative. Patients with ovarian cancer typically present with metastatic disease, with the most common site of metastasis being the peritoneum. Diagnosing ovarian cancer based on a metastatic inguinal lymph node is extremely rare (13). The literature reveals only a few cases where patients with ovarian carcinoma had positive inguinal lymph nodes at onset (14). Our patient initially sought medical attention for an enlarged right inguinal lymph node, and a diagnosis of high-grade serous carcinoma was made after its excision.

Metastasis to the inguinal region in ovarian cancer may occur through hematogenous spread or lymphatic spread via the round ligament (15). The lymphogenic-only spread in this case suggests a specific biological metastatic behavior. Some researchers proposed that host immune defense mechanisms may destroy the primary tumor without affecting lymphatic metastasis (16, 17), whereas others suggested that spread to the inguinal region might occur through the peritoneum after previous surgery (11), but our patient had no prior surgeries. Isolated lymph node disease in ovarian cancer appears to represent a less aggressive relapse pattern, associated with relatively mild behavior and few symptoms. According to some literature, this pattern may be related to the tumor cells’ low proliferation rate and initial lack of peritoneal spread (18). The lymph node microenvironment might keep cells dormant due to the presence of cytokines and T-cells (19). This metastatic pattern often shows higher lymphocyte infiltration, particularly of CD3 and CD8 cells, compared to extra-nodal ovarian cancer relapses. However, it does not show significant differences regarding known genomic subtypes, such as BRCA1/2 mutations or increased CCNE1 (20).

There are some limitations of the study. The current study is a single-case analysis, which restricts its broader applicability. Also, this case showed no germline or somatic BRCA1/2 mutations, and the homologous recombination deficiency (HRD) score was 53.44 (threshold value: 30). Homologous recombination deficiency may be one of the factors associated with isolated lymph node metastasis. Therefore, further research is needed to identify other genetic factors linked to this phenomenon. Specifically, the reasons for the left fallopian tube tumor presenting with right inguinal lymph node enlargement and the explanation for lymphatic drainage from the primary tumor to only the right inguinal lymph node require urgent investigation. Previous research suggests that the prognosis for this type of primary fallopian tube carcinoma (PFTC) appears to be good (14). Consequently, the patient will be followed up for a long time for clinical outcome.

## Conclusions

The literature reports a rare case of isolated ovarian metastases to the inguinal lymph nodes, and the mechanism by which fallopian tube cancer metastasizes to the contralateral groin lymph nodes remains unclear. Homologous recombination deficiency may play an important role in this type of primary fallopian tube carcinoma (PFTC). Therefore, patients like the one in this case may benefit from PARP inhibitor (PARPi) therapy.

## Data Availability

The original contributions presented in the study are included in the article/supplementary material. Further inquiries can be directed to the corresponding authors.

## References

[1] Sung H Ferlay J Siegel RL Laversanne M Soerjomataram I Jemal A . Global cancer statistics 2020: GLOBOCAN estimates of incidence and mortality worldwide for 36 cancers in 185 countries. CA Cancer J Clin. (2021) 71:209–49. doi: 10.3322/caac.21660 33538338

[2] Han X Wang Z Huang D Deng K Wang Q Li C . Analysis of the disease burden trend of Malignant tumors of the female reproductive system in China from 2006 to 2020. BMC Womens Health. (2022) 22:504. doi: 10.1186/s12905-022-02104-2 36476597 PMC9730658

[3] Rose PG Piver MS Tsukada Y Lau TS . Metastatic patterns in histologic variants of ovarian cancer. autopsy study. Cancer. (1989) 64:1508–13. doi: 10.1002/1097-0142(19891001)64:7<1508::AID-CNCR2820640725>3.0.CO;2-V 2776109

[4] Panici PB Angioli R . Role of lymphadenectomy in ovarian cancer. Best Pract Res Clin Obstet Gynaecol. (2002) 16:529–51. doi: 10.1053/beog.2002.0301 12413933

[5] Vizzielli G Costantini B Tortorella L Petrillo M Fanfani F Chiantera V . Influence of intraperitoneal dissemination assessed by laparoscopy on prognosis of advanced ovarian cancer: an exploratory analysis of a single-institution experience. Ann Surg Oncol. (2014) 21:3970–7. doi: 10.1245/s10434-014-3783-6 24849521

[6] Fournier M Stoeckle E Guyon F Brouste V Thomas L MacGrogan G . Lymph node involvement in epithelial ovarian cancer: sites and risk factors in a series of 355 patients. Int J Gynecol Cancer. (2009) 19:1307–13. doi: 10.1111/IGC.0b013e3181b8a07c 20009882

[7] Ulker V Kuru O Numanoglu C Akbayır O Polat I Uhri M . Lymph node metastasis in patients with epithelial ovarian cancer macroscopically confined to the ovary: review ofa single-institution experience. Arch Gynecol Obstet. (2014) 289:1087–92. doi: 10.1007/s00404-013-3078-3 24213097

[8] Benedetti-Panici P Greggi S Maneschi F Scambia G Amoroso M Rabitti C . Anatomical and pathological study of retroperitoneal nodes in epithelial ovarian cancer. Gynecol Oncol. (1993) 51:150–4. doi: 10.1006/gyno.1993.1263 8276287

[9] Maggioni A Benedetti Panici P Dell’Anna T Landoni F Lissoni A Pellegrino A . Randomised study of systematic lymphadenectomy in patients with epithelial ovarian cancer macroscopically confined to the pelvis. Br J Cancer. (2006) 95:699–704. doi: 10.1038/sj.bjc.6603323 16940979 PMC2360519

[10] Colombi I D'Indinosante M Lazzeri L Zupi E Pisaneschi S Giusti M . Tubal cancer clinical management: two exceptional scenarios and a review of the literature. J Clin Med. (2024) 13:5075. doi: 10.3390/jcm13175075 39274288 PMC11395798

[11] Metwally IH Zuhdy M Hassan A Alghandour R Megahed N . Ovarian cancer with metastatic inguinal lymphadenopathy: A case series and literature review. J Egypt Natl Canc Inst. (2017) 29:109–14. doi: 10.1016/j.jnci.2017.01.003 28258913

[12] Kleppe M Kraima AC Kruitwagen RF Van Gorp T Smit NN van Munsteren JC . Understanding lymphatic drainage pathways of the ovaries to predict sites for sentinel nodes in ovarian cancer. Int J Gynecol Cancer. (2015) 25:1405–14. doi: 10.1097/IGC.0000000000000514 PMC5106084 26397066

[13] Giancontieri P Turetta C Barchiesi G Pernazza A Pignataro G D’Onghia G . High-grade serous carcinoma of unknown primary origin associated with STIC clinically presented as isolated inguinal lymphadenopathy: a case report. Front Oncol. (2024) 13:1307573. doi: 10.3389/fonc.2023.1307573 38370346 PMC10870410

[14] Maeda M Hisa T Matsuzaki S Ohe S Nagata S Lee M . Primary fallopian tube carcinoma presenting with a massive inguinal tumor: A case report and literature review. Medicina. (2022) 58:581. doi: 10.3390/medicina58050581 35629998 PMC9147285

[15] Ang D Ng KY Tan HK Chung AY Yew BS Lee VK . Ovarian carcinoma presenting with isolated contralateral inguinal lymph node metastasis: a case report. Ann Acad Med Singap. (2007) 36:427–30. doi: 10.47102/annals-acadmedsg. 17597969

[16] Kehoe S Luesley D Rollason T . Ovarian carcinoma presenting with inguinal metastatic lymphadenopathy 33 months prior to intraabdominal disease. Gynecol Oncol. (1993) 50:128–30. doi: 10.1006/gyno.1993.1177 8349155

[17] Preston CC Goode EL Hartmann LC Kalli KR Knutson KL . Immunity and immune suppression in human ovarian cancer. Immunotherapy. (2011) 3:539–56. doi: 10.2217/imt.11.20 PMC3147144 21463194

[18] Restaino S Mauro J Zermano S Pellecchia G Mariuzzi L Orsaria M . CUP-syndrome:Inguinal high grade serous ovarian carcinoma lymph node metastases with unknown primary origin – A case report and literature review. Front Oncol. (2022) 12:987169. doi: 10.3389/fonc.2022.987169 36300091 PMC9589412

[19] Legge F Petrillo M Adamo V Pisconti S Scambia G Ferrandina G . Epithelial ovarian cancer relapsing as isolated lymph node disease: natural history and clinical outcome. BMC Cancer. (2008) 8:367. doi: 10.1186/1471-2407-8-367 19077269 PMC2632673

[20] Hollis RL Carmichael J Meynert AM Churchman M Hallas-Potts A Rye T . Clinical and molecular characterization of ovarian carcinoma displaying isolated lymph node elapse. Am J Obstet Gynecol. (2019) 221:245.e1–245.e15. doi: 10.1016/j.ajog.2019.04.035 PMC6857430 31055034

